# Untargeted analysis of plasma samples from pre-eclamptic women reveals polar and apolar changes in the metabolome

**DOI:** 10.1007/s11306-019-1600-8

**Published:** 2019-11-27

**Authors:** Katrin N. Sander, Dong-Hyun Kim, Catharine A. Ortori, Averil Y. Warren, Uchenna C. Anyanwagu, Daniel P. Hay, Fiona Broughton Pipkin, Raheela N. Khan, David A. Barrett

**Affiliations:** 1 Division of Medical Science and Graduate Entry Medicine, School of Medicine, University of Nottingham, Royal Derby Hospital, Uttoxeter Road, Derby, DE22 3DT UK; 2 Division of Child Health, Obstetrics & Gynaecology, School of Medicine, University of Nottingham, City Hospital, Nottingham, NG5 1PB UK; 3 Centre for Analytical Bioscience, School of Pharmacy, University of Nottingham, Nottingham, NG7 2RD UK

**Keywords:** Pregnancy, human, Pre-eclampsia, Metabolomics, Metabolic profiling

## Abstract

**Introduction:**

Pre-eclampsia is a hypertensive gestational disorder that affects approximately 5% of all pregnancies.

**Objectives:**

As the pathophysiological processes of pre-eclampsia are still uncertain, the present case–control study explored underlying metabolic processes characterising this disease.

**Methods:**

Maternal peripheral plasma samples were collected from pre-eclamptic (n = 32) and healthy pregnant women (n = 35) in the third trimester. After extraction, high-resolution mass spectrometry-based untargeted metabolomics was used to profile polar and apolar metabolites and the resulting data were analysed via uni- and multivariate statistical approaches.

**Results:**

The study demonstrated that the metabolome undergoes substantial changes in pre-eclamptic women. Amongst the most discriminative metabolites were hydroxyhexacosanoic acid, diacylglycerols, glycerophosphoinositols, nicotinamide adenine dinucleotide metabolites, bile acids and products of amino acid metabolism.

**Conclusions:**

The putatively identified compounds provide sources for novel hypotheses to help understanding of the underlying biochemical pathology of pre-eclampsia.

**Electronic supplementary material:**

The online version of this article (10.1007/s11306-019-1600-8) contains supplementary material, which is available to authorized users.

## Introduction

Pre-eclampsia is a syndrome that complicates between 3 and 5% of pregnancies (Lisonkova and Joseph 2013). It is defined by de novo hypertension with proteinuria and directly implicated in 14% of global maternal deaths, preterm deliveries and infants of low-birthweight (Steegers et al. 2010; WHO 2015). The impact of this disease is far-reaching with a significantly increased risk of diabetes and cardiovascular disease in later life (Murphy and Smith 2016).

The pathophysiology of pre-eclampsia has been the subject of substantial research efforts in past decades (Huppertz 2008). There is a general consensus that the maternal pre-eclamptic syndrome is caused by maternal systemic endothelial dysfunction triggered by pregnancy (Redman 1991). In the first stage, inadequate villous trophoblast differentiation is seen as the origin of pre-eclampsia. The second (symptomatic) stage proposes a state in which the mother, due to genetic or environmental factors or increased placental mass, is not able to cope with the placental release of apoptotic microvesicles (Huppertz 2008).

Insufficient insight into the pathophysiology of pre-eclampsia is a major reason for the lack of early diagnostic markers, hampering the reliable diagnosis or evaluation of disease progression essential for the management of this potentially life-threatening gestational complication. Therefore, increasing efforts aim to understand perturbed metabolic pathways, both as potential sources of early biomarkers and to uncover metabolic processes involved in the pathophysiology of pre-eclampsia. In this context, the discovery of early biomarkers is an important aim for diagnostic purposes. However, an examination of the metabolome during the clinical phase of pre-eclampsia is also crucial to understand ongoing changes in metabolic processes during the symptomatic phase of the disease. Most of the previously suggested biomarkers for pre-eclampsia are proteins, presumed to be derived from the placenta or damaged vascular endothelium (Wu et al. 2015). Evaluation of their sensitivity and specificity was inconclusive with more studies needed to confirm their utility in the clinic.

A number of authors have previously searched for biomarkers in maternal blood in pre-eclampsia using metabolomics. These studies assessed maternal plasma (Bahado-Singh et al. 2012; De Oliveira et al. 2012; Kenny et al. 2005, 2008; Kenny et al. 2010; Turner et al. 2008) or serum (Bahado-Singh et al. 2013; Kuc et al. 2014; Odibo et al. 2011) collected before (first or second trimester) (Bahado-Singh et al. 2012, 2013; Kenny et al. 2010; Kuc et al. 2014; Odibo et al. 2011) and/or after (De Oliveira et al. 2012; Kenny et al. 2005, 2008; Turner et al. 2008) disease onset. Half of the studies distinguished between early and late onset pre-eclampsia (Bahado-Singh et al. 2012, 2013; De Oliveira et al. 2012; Kuc et al. 2014), the other half used sample sets without considering the gestation at the onset of the disease (Kenny et al. 2005, 2008, 2010; Odibo et al. 2011; Turner et al. 2008). Analyses were performed with sample numbers ranging from 8 to 100 in the pre-eclampsia group (Bahado-Singh et al. 2012, 2013; De Oliveira et al. 2012; Kenny et al. 2005, 2008, 2010; Kuc et al. 2014; Odibo et al. 2011; Turner et al. 2008). A range of metabolites including polar and apolar compounds have been proposed as biomarkers. The most comprehensive study in this context utilised a validated untargeted analysis using LC–MS with approximately 100 patients in each subject group (Kenny et al. 2010): It was suggested that a combination of 14 metabolites could predict pre-eclampsia before the onset of the disease.

However, a recent systematic review and meta-analysis concluded that “although there are multiple potential biomarkers for PE their efficacy has been inconsistent”, (Wu et al. 2015). This confusion is likely to be caused by the different experimental (chemical and instrumental) approaches and clinical designs chosen and highlights the need for additional studies in this field. Confirmation of a specific metabolic pathway would set the basis for future studies focussing on specific substance groups.

Although early markers are important, metabolic changes are most likely to be maximal after onset of the disease. As a prelude to the application of metabolomics in identifying metabolites associated with the pre-eclamptic syndrome, the present study was designed to test plasma from women already diagnosed with pre-eclampsia to provide proof of concept that the metabolome of pre-eclamptic women is distinct from that of women with a normal uncomplicated pregnancy. For this, a comprehensive analytical approach was chosen to avoid focussing on a specific group of  metabolites.

## Materials and methods

### Chemicals

Acetonitrile, chloroform and ammonium acetate were purchased from Fisher Scientific (Loughborough, UK). Ammonium carbonate, 2-propanol and methanol were purchased from Fluka (Sigma-Aldrich, Hannover, Germany). Deionized water (18.2 MΩ) was prepared using an ELGA USF-Maxima water purification system (Marlow, UK).

### Plasma collection and preparation

Maternal peripheral blood samples were taken at Royal Derby Hospital (UK) from healthy (n = 35) or pre-eclamptic (n = 32) pregnant women after obtaining fully informed written consent. Ethics approval for the sample collection and utilisation was granted by Derbyshire Research Ethics Committee (REC Reference No. 09/H0401/90). Recruited subjects in the pre–eclampsia group had (1) normal booking blood pressures (at 12–20 weeks of pregnancy), (2) subsequently developed blood pressures of ≥ 140 systolic or ≥ 90 diastolic on two occasions (minimum 24 h apart) and (3) had at least 1+ proteinuria (≥ 300 mg/L) using dipstick analysis (Meyer et al. 1994). These criteria correspond to current guidelines for diagnosis of pre–eclampsia (Magee et al. 2014). Subjects in the control group did not have any documented hypertensive problems throughout their pregnancy. Pre-eclampsia and control samples were matched for age, BMI, systolic booking blood pressure, gestational age at sampling, parity and ethnicity. Patients were not matched for diastolic booking blood pressure, gestational age at delivery, birth weight/customized weight centile of the baby or multiple pregnancy. Patient demo-graphics are shown in Table 1. Early onset pre–eclampsia affected 10 of the 32 pre-eclampsia patients.

**Table 1:** Patient demographics for maternal plasma collected from control and pre-eclampsia cohort

		Control	Pre-eclampsia	p value
		(n = 35)	(n = 32)	
Age (yrs)		28.9 (6.7)	30.2 (5.1)	> 0.05
BMI		27.0 (7.0)	28.0 (5.1)	> 0.05
Booking blood pressure systolic (mmHg)		109.4 (8.4)	112.6 (9.1)	> 0.05
Booking blood pressure diastolic (mmHg)		63.6 (7.4)	69.8 (8.7)	0.003
Gestational age at sampling (wks)		36.7 (3.2)	35.9 (2.8)	> 0.05
Gestational age at delivery (wks)		38.3 (2.0)	36.9 (2.6)	0.016
Birth weight baby (g)		3149.1 (698.9)	2543.6 (830.7)	0.002
Customised weight centile baby		48.2 (30.0)	27.9 (31.0)	0.008
Multiple pregnancy	Singleton	35 (100%)	28 (87.5%)	0.031
	Twins	0	4 (12.5%)	
Parity	0	14 (40.0%)	22 (68.8%)	> 0.05
	1	13 (37.1%)	5 (15.6%)	
	2	4 (11.4%)	4 (12.5%)	
	3	3 (8.6%)	1 (3.1%)	
	4	1 (2.9%)	0 (0.0%)	
Ethnicity	Africa	0 (0.0%)	1 (3.1%)	> 0.05
	India/Pakistan	4 (11.4%)	4 (12.5%)	
	Middle East	0 (0.0%)	1 (3.1%)	
	Southern/Northern Europe	31 (88.6%)	26 (81.3%)	

Table shows mean (standard deviation) or numbers (%) of n recruited patients. *yrs* years, *wks* weeks. Customised weight centiles were calculated using Weight Centile Calculator from GROW software version 6.7.7, 2016 (Gardosi et al. 1992, 1995). Significance testing was performed using student t-test (continuous variables) or Chi square test (categorical variables)

### Plasma preparation

Blood samples were taken into BD Vacutainer EDTA blood collection tubes and centrifuged at 1000×*g* for 15 min at 4 °C. The plasma was transferred to Eppendorf tubes in 0.5 mL aliquots, snap-frozen and stored at − 80 °C. Only samples that were processed within 1 h were included in this study.

### Metabolite extraction

Polar and apolar metabolites were extracted from plasma samples (400 µL) with 500 µL chloroform:methanol (1:2) followed by centrifugation at 13,000×*g* for 10 min at 4 °C. 75 µL of the lower chloroform (apolar) phase were added to prepared vials containing 1500 µL isopropanol, giving the readily injectable apolar fraction. 600 µL of the aqueous (polar) supernatant were transferred into Eppendorf tubes and mixed with 900 µL acetonitrile to precipitate proteins. Samples were centrifuged at 13,000×*g* for 10 min at 4 °C and 1400 µL were transferred into Eppendorf tubes. Following solvent evaporation, the residue was resuspended in 100 µL starting mobile phase (H_2_O:acetonitrile 4:6) prior to injection. A pooled quality control (QC) sample was prepared for each fraction. This was achieved by pooling a 10 µL aliquot of each extract of the respective fraction.

### LC–MS polar fraction

The LC–MS method was based on a previously reported method (Kim et al. 2014). 10 µL of the polar fraction of each sample were injected onto an Exactive high-resolution mass spectrometry (HRMS) system operating in positive and negative ion mode (Accela U-HPLC system coupled to a HESI-II Exactive Orbitrap mass spectrometer, Thermo Fisher, Hemel Hempstead, UK). Chromatographic separation was achieved using a Sequant ZIC-pHILIC 5 µm PEEK 150 × 4.6 mm column (Merck Millipore, Darmstadt, Germany) using a gradient over 15 min (mobile phase A: 0 min 40%; 9 min 95%; 10 min 40%; 15 min 40% at 300 µL/min). Mobile phase A was H_2_O with 20 mM (NH_4_)_2_CO_3_ and mobile phase B consisted of acetonitrile without additive. Following interface parameters were used: spray voltage 4500 V (positive mode)/3500 V (negative mode); capillary temperature 275 °C; heater temperature 150 °C; flow rates of sheath, auxiliary and sweep gas were 40, 5 and 1 (arbitrary units); capillary, tube and skimmer voltages were − 28.6 V, − 67.5 V and − 16.6 V. Data acquisition was performed in full scan mode with a range from *m/z* 70 to 1400.

### LC–MS apolar fraction

The LC–MS/MS method was based on a previously reported method (Ravipati et al. 2015). 10 µL of the apolar fraction of each sample were injected onto an Exactive HRMS system operating in positive and negative ion mode. Chromatographic separation was achieved using a Poroshell SBC18 50 × 2.1 mm 2.7 µm column (Agilent, Santa Clara, US) using a gradient over 12 min (mobile phase B: 0 min 32%; 1 min 60%; 5 min 75%; 6 min 100%; 10 min 100%; 11 min 32%; 12 min 32% at 450 µL/min). Mobile phase A consisted of 40% acetonitrile and 60% H_2_O with 13 mM NH_4_Ac. Mobile phase B consisted of 10% acetonitrile, 10% H_2_O and 80% isopropanol with 13 mM NH_4_Ac. Following interface parameters were used in both negative and positive mode: spray voltage 4000 V; capillary temperature 250 °C; heater temperature 300 °C; flow rates of sheath, auxiliary and sweep gas were 30, 15 and 5 (arbitrary units); capillary, tube and skimmer voltages were − 26.0 V, − 137.0 V and − 26.6 V. Data acquisition was performed in full scan mode with a range from *m/z* 100 to 1900.

### LC–MS analysis

Samples for polar and apolar fraction were analysed in separate runs according to a run design as suggested previously with QC and samples analysed after every 11 samples (Zelena et al. 2009). Samples were randomized before extraction and before injection. Pooled QC samples were used to assess the analytical performance.

### LC–MS data analysis

Generated raw data was acquired using Xcalibur software (Thermo Scientific, Hemel Hempstead, UK). The data was pre-processed using Progenesis QI software (Nonlinear Dynamics, Newcastle upon Tyne, UK). Peak picking was performed with auto threshold and an automatically chosen QC injection served as reference for chromatographic alignment and normalisation. Peak intensities were normalised to all compounds. For this, abundance ratios between each sample and the reference run are calculated for all compounds. Log10 ratio distributions of each sample are then centred onto the log10 ratio distribution of the reference run by applying an additive or subtractive shift in the log space (Waters Corporation 2015). For further advanced multivariate analysis (MVA), the four datasets were merged to one large dataset before importing to SIMCA (Version 13.0, Umetrics AB, Umea, Sweden). The data was scaled in the default setting unit variance (UV) and log transformation was applied. PCA and OPLS-DA models of the data were created. Cross validation was performed by creating an OPLS-DA model with a portion of the observations to avoid potential bias. The remaining observations were subsequently fitted into this model. In the resulting model, variables with a VIP value > 1 were identified as potential biomarkers.

Univariate analysis (UVA) was performed using Progenesis QI. Criteria for potential biomarkers were a QC CV ≤ 30%, a fold intergroup change of ≥ 1.5 and significantly changed metabolite levels after *t* test and correction for multiple testing using FDR (false discovery rate). Regression analysis was performed to evaluate the effects of two unmatched patient parameters (diastolic booking blood pressure and singleton/multiple pregnancy) as potential confounders. Other unmatched patient parameters (gestational age at delivery and birth weight/customized weight centile of the baby) were not tested as these parameters have a causal relationship with pre-eclampsia and with that do not meet the criteria for a confounding factor.

Biomarkers that were filtered by both UVA and MVA were putatively identified by searching for their *m/z* values in the Human Metabolome Database (www.hmdb.ca), METLIN Metabolomics Database (www.metlin.scripps.edu) and Lipid Maps (www.lipidmaps.org). Putative identifications were based on isotope similarity > 75%, mass error < 1.5 ppm and expected or demonstrated presence in the human organism. Metabolite identification confidence was classified using identification classes (Sumner et al. 2007). Additionally, the quantitative identification score was determined as proposed previously (Sumner et al. 2014).

## Results

### Metabolomic analysis of plasma from control and pre-eclamptic subjects

Plasma samples from pregnant women with (n = 32) and without (n = 35) pre-eclampsia were analysed in this study to search for metabolites indicating altered biological pathways involved in the pathophysiology of pre-eclampsia.

LC–MS metabolomics quality control (QC) samples met the required acceptability threshold for both the polar and apolar metabolomics analysis methods used in the study (Chan et al. 2011; Dunn et al. 2011). Assessment of the peak areas of key ions of QC samples confirmed analytical stability in terms of peak area (< 30% relative standard deviation (RSD)) and retention time (< 2% RSD).

Initial processing of the metabolomics data from the study samples revealed 4379 peaks of polar compounds and 3153 peaks of apolar compounds detected by LC–MS. MVA and UVA of these data sets was used to define a set of metabolites which showed significant differences between the control and pre-eclampsia study groups.

**Fig. 1:**
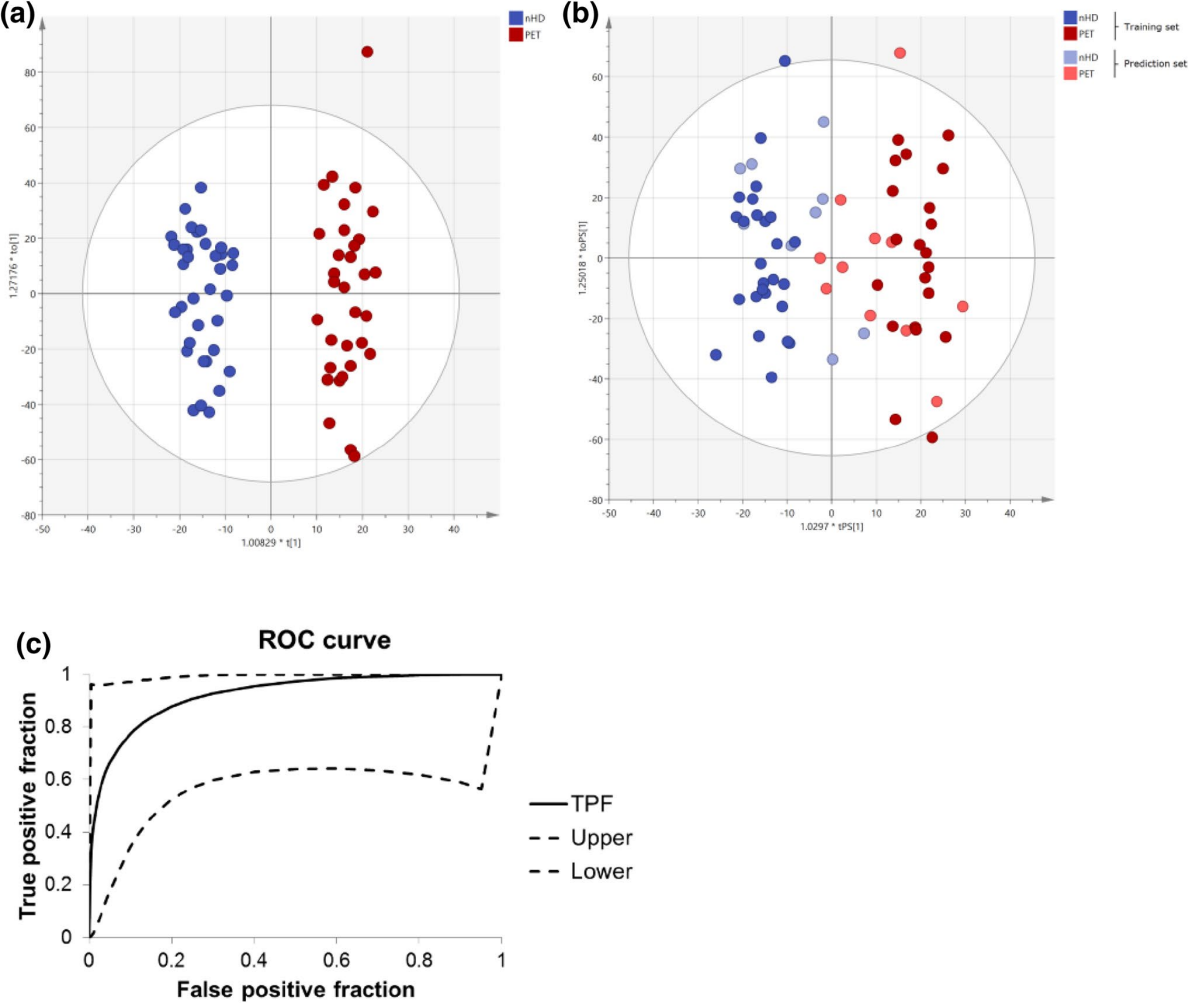
Multivariate analysis based on all detected ions: **a** OPLS-DA score plot of control (no hypertensive disease, nHD, n = 35) and pre-eclampsia (PET, n = 32) samples for all variables. **b** OPLS-DA score plot of training and prediction set for control (no hypertensive disease, nHD) and pre-eclampsia (PET) samples for all variables. **c** Receiver operator characteristic (ROC) curves for all variables. The figure shows the true positive fraction (TPF) with upper and lower 95% confidence intervals. The AUC is 0.922 with a standard error of 0.06

MVA showed no visible separation of control and pre-eclampsia subjects in a principal component analysis (PCA) (data not shown). However, the orthogonal projections to latent structures discriminant analysis (OPLS-DA) model in Fig. 1a shows a complete separation of control subjects and pre-eclampsia subjects. The R2X(cum)-value of 0.286 and Q2(cum)-value of 0.514 are typical for clinical samples. Furthermore, the CV-ANOVA p value is highly significant (p = 6 × 10^−7^), indicating a good model. Validation of the dataset was performed by randomly choosing 30% of the initial observations and creating a model with the remaining observations. The new model based on these training and prediction data sets is shown in Fig. 1b. Class affiliation of previously removed observations was then predicted. 85% of the observations were predicted correctly with a sensitivity of 82% and specificity of 89%. This demonstrated good quality of the OPLS-DA training model, as a prediction of those observations that were left out would not be correct in an over-fitted model. Figure 1c shows a receiver operator characteristic (ROC) curve to evaluate the predictability of the OPLS-DA model shown in Fig. 1b (Eng 2007). The area under the curve was 0.922.

### Putative plasma biomarkers of pre-eclampsia

To identify biomarkers for pre-eclampsia, detected compounds were further filtered. Univariate analysis (UVA) yielded 37 metabolites after significance testing using t-test and correction for multiple testing. Based on a variable influence on projection (VIP) value > 1 in the cross validated OPLS-DA model shown in Fig. 1b, 2527 ions were filtered using multivariate analysis (MVA). All 37 compounds filtered by UVA were also filtered by MVA and are therefore promising candidates for biomarkers (see full list in the Supplemental data Table 1).

Two of the unmatched patient parameters were classified as potential confounders: (1) diastolic booking blood pressure and (2) singleton/multiple pregnancy. Regression analysis showed that none of these parameters were statistically significant (independent) confounders (data not shown). Therefore, the data was not adjusted for these parameters.

**Fig. 2:**
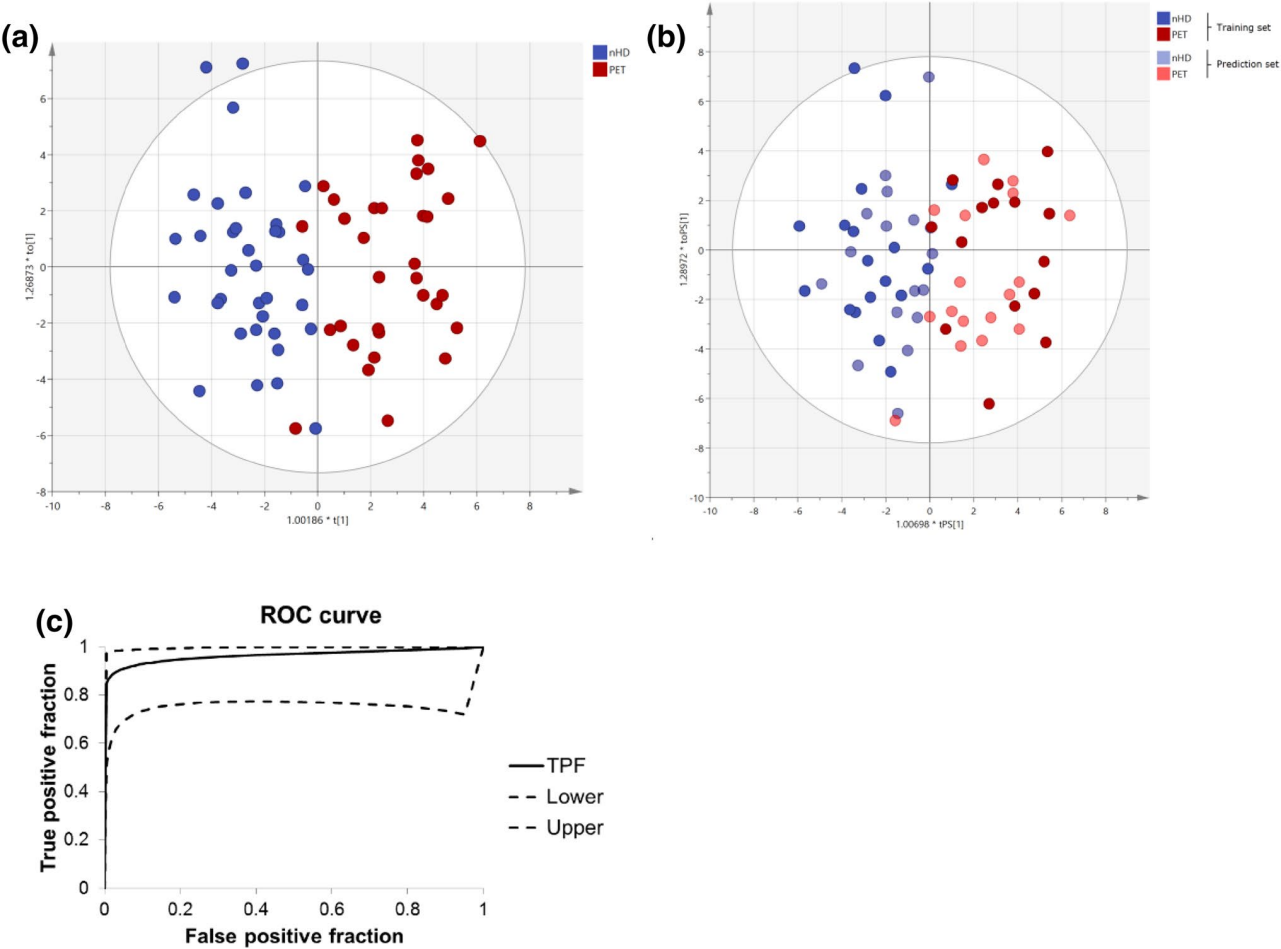
Multivariate analysis based on the 35 most predictive ions: **a** OPLS-DA score plot of control (no hypertensive disease, nHD, n = 35) and pre-eclampsia (PET, n = 32) samples for the 35 most predictive ions. **b** OPLS-DA score plots of training and prediction set for control (no hypertensive disease, nHD) and pre-eclampsia (PET) samples for the 35 most predictive ions. **c** Receiver operator characteristic (ROC) curves for the 35 most predictive ions. The figure shows the true positive fraction (TPF) with upper and lower 95% confidence intervals. The AUC is 0.964 with a standard error of 0.04

Multivariate analysis was repeated based on the putative biomarker metabolites. Two compounds were removed from this list and all further analyses, as these were putatively identified as labetalol and terbutaline—medications commonly given to pre-eclamptic women. The PCA based on the 35 most predictive ions showed a visible separation of control and pre-eclampsia subjects (data not shown). Similarly, the OPLS-DA model in Fig. 2a shows a complete separation of control subjects and pre-eclampsia subjects. The R2X(cum)-value of 0.629, Q2(cum)-value of 0.648 and a highly significant CV-ANOVA p value (p = 5.5 × 10^−12^) indicate an excellent model. A validation of the dataset was performed by randomly choosing 50% of the initial observations and creating a model with the remaining observations. The new model based on these training and prediction data sets is shown in Fig. 2b. The class affiliation of previously removed observations was then predicted. 91% of the observations were predicted correctly with a sensitivity of 82% and specificity of 100%, demonstrating excellent quality of the OPLS-DA training model. Figure 2c shows a ROC curve to evaluate the predictability of the OPLS-DA model shown in Fig. 2b (Eng 2007). The area under the curve was 0.964.

**Fig. 3:**
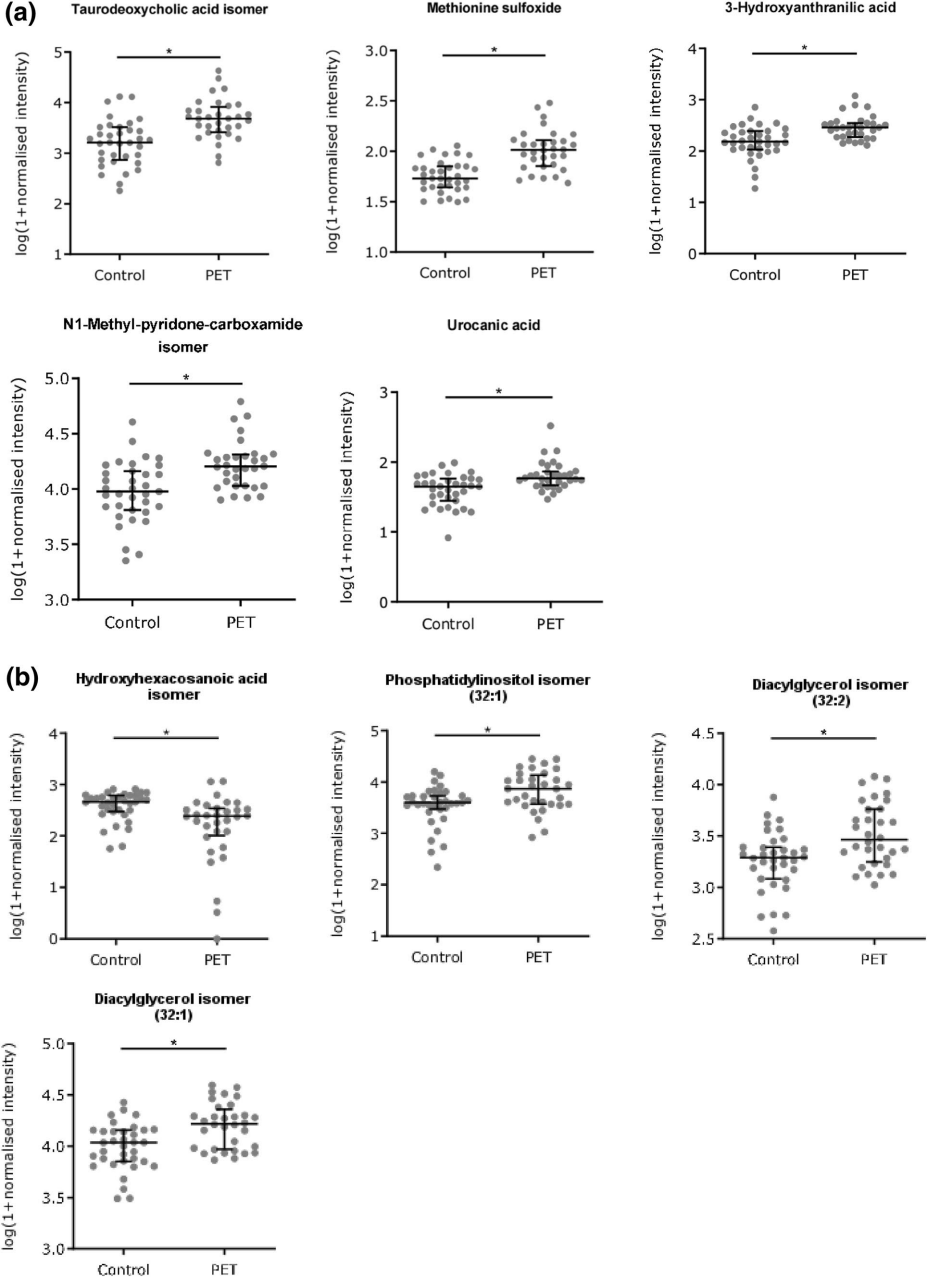
Scatter dot plots of potential biomarkers in the polar (**a**) and apolar (**b**) phase. All shown biomarkers fulfil criteria of both MVA and UVA. MVA criteria were a VIP value > 1 in the OPLS-DA model shown in Fig. 1c. Criteria for UVA were a q-value < 0.05 (*), a fold change > 1.5 and CV(QC) < 30%. Dots show log(1 + normalised intensity) of control (n = 35) and pre-eclampsia (PET, n = 32) samples. Error bars represent median and interquartile ranges. For more details see supplemental data (Tables 1, 2 and Table 3)

Using metabolomics databases, 9 of the 35 compounds could be putatively identified by compound class or unique assignment (see Supplemental data Tables 2 and 3): A taurodeoxycholic acid isomer, methionine sulfoxide, 3-hydroxyanthranilic acid, an N1-methyl-pyridone-carboxamide isomer and urocanic acid in the polar fraction and a hydroxyhexacosanoic acid isomer, two different types of diacylglycerols and a glycerophosphoinositol in the apolar fraction. Scatter plots of control vs PET normalised intensities for these compounds are shown in Fig. 3. Identification classes (Sumner et al. 2007) are shown in Supplemental data Tables 2 and 3. The quantitative identification score was 1.0 in all cases (Sumner et al. 2014).

## Discussion

The present study examined the metabolome of maternal plasma samples to identify altered metabolite pathways that may provide new insights into the underlying biology of pre-eclampsia. This is the first report of a separate analysis of polar and apolar metabolites in plasma samples for a metabolomics approach in this disorder. This comprehensive analytical approach leads to an improved resolution of the metabolome into its distinct components resulting in maximum coverage of the metabolome in the biological sample.

This study included patients with mixed risk factors for pre-eclampsia. The sample set for pre-eclampsia included subjects of mixed parity, women with early and late onset pre-eclampsia as well as single and multiple pregnancies. Parity and multiple pregnancy are important risk factors for pre-eclampsia. Furthermore early and late onset pre-eclampsia are increasingly seen as two different disease entities (Raymond and Peterson 2011). The biomarkers identified in this pilot work are therefore mainly informative for ongoing processes during the clinical appearance of the pre-eclamptic syndrome, which is a common feature of pre-eclampsia independent of its origin.

A total of 35 metabolites were found to significantly contribute to metabolic differences in plasma from pre-eclamptic and healthy pregnant women. Not all of these could be assigned chemical identities, but the use of database matching allowed putative identification of several metabolites.

The two N-methylpyridone carboxamide isomers are NAD metabolites and have previously been shown to accumulate in patients with renal failure (Rutkowski et al. 2003). Levels were increased in plasma from pre-eclamptic women. They are of interest given that pre-eclampsia is characterised by proteinuria, an important diagnostic marker for underlying renal dysfunction. Metabolic markers may be superior to proteinuria as a diagnostic tool, as the reliability of the latter is under scrutiny (Magee et al. 2014).

A further set of metabolites showing increased levels in our pre-eclampsia study was assigned to three closely related bile acids: tauro-deoxycholic acid, taurourso-deoxycholic acid and taurocheno-deoxycholic acid. Taurine, an element of the three structural isomers, has previously been found as a biomarker in other studies analysing maternal blood in pre-eclampsia (Kenny et al. 2010; Kuc et al. 2014).

A previously proposed marker for oxidative stress, methionine sulfoxide, was increased in pre-eclampsia (Mashima et al. 2003; Raff et al. 2008). This is in line with the increased placental oxidative stress as a known feature in pre-eclampsia. Interestingly, levels of the antioxidant 3-hydroxyanthranilic acid were also raised in pre-eclamptic subjects. It is an intermediate of tryptophan catabolism via the kynurenine pathway, which is an important pathway for the synthesis of NAD. The kynurenine pathway including 3-hydroxyanthranilic acid as a therapeutic target in pre-eclampsia was previously hypothesised but further evidence is required (Worton et al. 2019). An earlier study looking at kynurenine pathway metabolites identified kynurenic acid as a plasma marker for pre-eclampsia, however, in contrast to our study no significant changes were seen for 3–hydroxyanthranilic acid (Nilsen et al. 2012).

Levels of the histidine metabolite urocanic acid were found to be elevated in plasma from pre-eclamptic subjects. Urocanic acid is mainly known for its role as photoprotectant in the skin, however, it was also identified in amniotic fluid from normal pregnancies (Orczyk-Pawilowicz et al. 2016).

Concentrations of several lipids were found to change significantly when comparing the control and pre-eclampsia group. The concentration of the oxylipin, hydroxyhexacosanoic acid, was found to be decreased in plasma from pre-eclamptic women. Its dehydroxylated form, hexacosanoic acid, was earlier listed as biomarker in pre-eclampsia (Kenny et al. 2010). The complex changes in the oxylipin metabolism are likely to cause substantial physiological effects considering the potency of this molecule class in the regulation of vascular tone and PPAR related gene expression (Omar et al. 1992; Shiraki et al. 2005). For three further lipids a specific identification was not possible but they could be assigned to the lipid classes of diacylglycerol and glycerophosphoinositol isomers. These molecules are part of cell membranes and play a role in signal transduction and fatty acid storage (Romanowicz and Bankowski 2009).

Two additional compounds were putatively identified as the exogenous substances labetalol and terbutalin. Labetalol is used for the treatment of hypertension in pregnant women and a number of subjects in the pre-eclampsia cohort were receiving this drug. Terbutalin is a β_2_-receptor agonist, used for the management of asthma symptoms or as a tocolytic. The fact that the platform could recognise these compounds provides confidence for the applicability of the experimental approach. It is important to note that metabolomics is sensitive to exogenous influences in that it will also detect exogenous compounds derived from diet, food, lifestyle, drugs or gut microflora.

In summary, this study demonstrates that the metabolome undergoes substantial changes in pre-eclamptic women and a range of novel biomarkers were identified. Of the aforementioned compounds none are known and clinically used biomarkers in pre-eclampsia (Magee et al. 2014). As such, the identified compounds represent novel biomarkers that could help to provide new insights into the ongoing pathophysiological processes of this disease and could eventually lead to the identification of new drug targets. Although most of the putatively identified biomarkers have not been previously described, it was in many cases possible to relate their metabolic pathways to previously published studies as described above.

This work illustrates the potential of untargeted metabolomics in pre-eclampsia research to discover metabolic perturbations in pre-eclampsia potentially leading to novel hypotheses to help understand the underlying aetiology of this disease.

## Electronic supplementary material

Below is the link to the electronic supplementary material.

Supplementary material 1 (DOCX 62 kb)

## Abbreviations

CV-ANOVA: ANOVA of the cross-validated residuals
FDR: False discovery rate
LC–MS: Liquid chromatography–mass spectrometry
MVA: Multivariate analysis
OPLS-DA: Orthogonal projections to latent structures discriminant analysis
PCA: Principle component analysis
PET: Pre-eclampsia (pre-eclamptic toxaemia)
QC: Quality control
ROC: Receiver operator characteristic
RSD: Relative standard deviation
UVA: Univariate analysis
VIP: Variable influence on projection
