# Supplementary material for: Untargeted analysis of plasma samples from pre-eclamptic women reveals polar and apolar changes in the metabolome
Source: Metabolomics. 2019 Nov 27;15(12):157. doi: 10.1007/s11306-019-1600-8 (PMC6879453; doi:10.1007/s11306-019-1600-8)
Supplement: Supplementary file 1 — Supplementary material 1 (DOCX 62 kb) [file 11306_2019_1600_MOESM1_ESM.docx]

## Supplemental data

Table 1: Key mass ions that could potentially serve as biomarkers for pre-eclampsia.^a^ Masses were filtered by UVA and MVA. Where more than one adduct was detected, the neutral mass of the compound was calculated. Putatively identified compounds are listed in Table 2 and Table 3.

^a^ Two compounds were removed from all further analyses, as these were putatively identified as Labetalol (*m/z* 329.186) and Terbutaline (*m/z* 208.133), medications commonly given to pre-eclamptic women.

Table 2: Putative identification of biomarkers for pre-eclampsia from the polar fraction. Metabolites that were filtered by both UVA and MVA were putatively identified based on their mass. Putative identification based on isotope similarity > 75%, mass error < 1.5ppm and biological relevance. Metabolite identification confidence levels (Sumner *et al.*, 2007) : Level 1: Identified metabolites; Level 2: Putatively annotated compounds; Level 3: Putatively characterised compound classes; Level 4: Unknown compounds. The quantitative identification score was 1.0 in all cases (Sumner *et al.*, 2014).

| ***m/z*** | **Adducts** | **Formula** | **Mass Error (ppm)** | **Isotope Similarity (%)** | **Putative identification** | **Description (HMDB)** | **Confidence level** | **Compound ID** |
| --- | --- | --- | --- | --- | --- | --- | --- | --- |
| 517.331 | M+NH4 | C_26_H_45_NO_6_S | 0.97 | 85.58 | Tauroursodeoxycholic acid, Taurodeoxycholic acid, Taurochenodesoxycholic acid | Taurinated bile acids | 2 | HMDB0000874; HMDB0000951; HMDB0000896 |
| 498.290 | M-H | C_26_H_45_NO_6_S | 0.14 | 89.79 |  |  |  |  |
| 166.053 | M+H | C_5_H_11_NO_3_S | -0.19 | 89.32 | Methionine sulfoxide | Biomarker for oxidative stress | 2 | HMDB02005 |
| 154.050 | M+H | C_7_H_7_NO_3_ | 0.39 | 91.71 | 3-Hydroxyanthranilic acid | Product of the tryptophan metabolism, proposed to be a free radical scavenger and carcinogen | 2 | HMDB0001476 |
| 153.066 | M+H | C_7_H_8_N_2_O_2_ | 0.70 | 91.71 | N1-Methyl-4-pyridone-3-carboxamide, or N1-methyl-2-pyridone-5-carboxamide | End products of NAD degradation and serum biomarker for renal failure | 2 | HMDB0004194 HMDB0004193 |
| 151.051 | M-H | C_7_H_8_N_2_O_2_ | -0.48 | 91.69 |  |  |  |  |
| 139.050 | M+H | C_6_H_6_N_2_O_2_ | 0.79 | 93.28 | Urocanic acid | Histidine metabolite | 2 | HMDB0000301 |

Table 3: Putative identification of biomarkers for pre-eclampsia from the apolar fraction. Metabolites that were filtered by both UVA and MVA were putatively identified based on their mass. Putative identification based on isotope similarity > 75%, mass error < 1.5ppm and biological relevance. Metabolite identification confidence levels (Sumner *et al.*, 2007) : Level 1: Identified metabolites; Level 2: Putatively annotated compounds; Level 3: Putatively characterised compound classes; Level 4: Unknown compounds. The quantitative identification score was 1.0 in all cases (Sumner *et al.*, 2014). DG: Diacylglycerol; PI: Glycerophosphoinositol.

| ***m/z*** | **Adducts** | **Formula** | **Mass Error (ppm)** | **Isotope Similarity (%)** | **Putative identification** | | **Description (HMDB)** | **Confidence level** | **Compound ID** |
| --- | --- | --- | --- | --- | --- | --- | --- | --- | --- |
| 411.384 | M-H | C_26_H_52_O_3_ | -1.49 | 77.69 | Hydroxyhexacosanoic acid isomer | | Oxylipin | 3 | n.a. |
| 807.503 | M-H | C_41_H_77_O_13_P | 0.52 | 90.46 | PI(32:1) | or isomers | Role as cell membrane component, fatty acid storage and in cell signalling | 3 | n.a. |
| 587.464 | M+NH4, M+Na | C_35_H_64_O_5_ | -1.22 | 78.37 | DG(32:2) | or isomers | Role as cell membrane component, fatty acid storage and in cell signalling | 3 | n.a. |
| 589.480 | M+NH4, M+Na | C_35_H_66_O_5_ | -1.15 | 90.01 | DG(32:1) | or isomers | Role as cell membrane component, fatty acid storage and in cell signalling | 3 | n.a. |
